# A Rare Extramedullary Presentation of Multiple Myeloma: Paraspinal Muscle Involvement Revealed by FDG PET/CT

**DOI:** 10.4274/tjh.galenos.2020.2020.0134

**Published:** 2021-02-25

**Authors:** Elgin Özkan, Mine Araz, Güldane Cengiz Seval, Demet Nak, Meral Beksaç

**Affiliations:** 1 Ankara University Faculty of Medicine, Department of Nuclear Medicine, Ankara, Turkey; 2 Ankara University Faculty of Medicine, Department of Hematology, Ankara, Turkey

**Keywords:** Myeloma and other plasma cell dyscrasias, Positron emission tomography, FDG

A 51-year-old male with recurrent multiple myeloma (MM) was referred for positron emission tomography with 2-deoxy-2-[fluorine-18]fluoro-D-glucose integrated with computed tomography (18F-FDG PET/CT) to evaluate response to chemotherapy. 18F-FDG PET/CT showed diffuse markedly increased uptake in the right paraspinal muscles (Figure 1). Recurrence of kappa light chain myeloma was confirmed with diffuse infiltration of clonal kappa-positive plasma cells in the bone marrow. Ultrasound-guided biopsy of the paraspinal muscles and cytological analysis of closed-needle pleural biopsy revealed kappa monotypic plasma cell infiltration (Figure 2). Although radiotherapy targeting the paraspinal area was initiated, he was lost due to deep vein thrombosis and pulmonary embolism before radiotherapy could be completed.

Plasmacytomas mostly occur within or adjacent to the bone, but can also be found in soft tissues [1]. However, isolated intramuscular manifestation of MM is rare [2,3]. If detected, the most common location of presentation is paraspinal and the thigh muscles, followed by the iliopsoas and calf muscles. [3]. Studies have shown that FDG PET/CT can detect a larger number of extramedullary plasmacytoma (EMP) sites compared to magnetic resonance imaging and it also has higher sensitivity and specificity for detecting EMP than intramedullary lesions in MM [3,4,5]. In this case, a rare presentation of extramedullary MM was successfully demonstrated by FDG PET/CT.

## Figures and Tables

**Figure 1:**
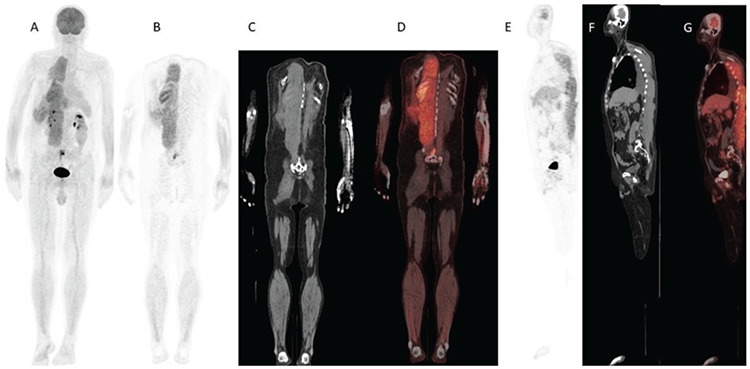
(A) Maximum intensity projection and (B) coronal PET, (C) coronal CT, (D) coronal fused and (E) sagittal PET, (F) sagittal CT, and (G) sagittal fused FDG PET/CT images demonstrate extensive and diffuse FDG uptake in paraspinal muscles. FDG: Fluorodeoxyglucose, PET/CT: positron emission tomography/computed tomography.

**Figure 2:**
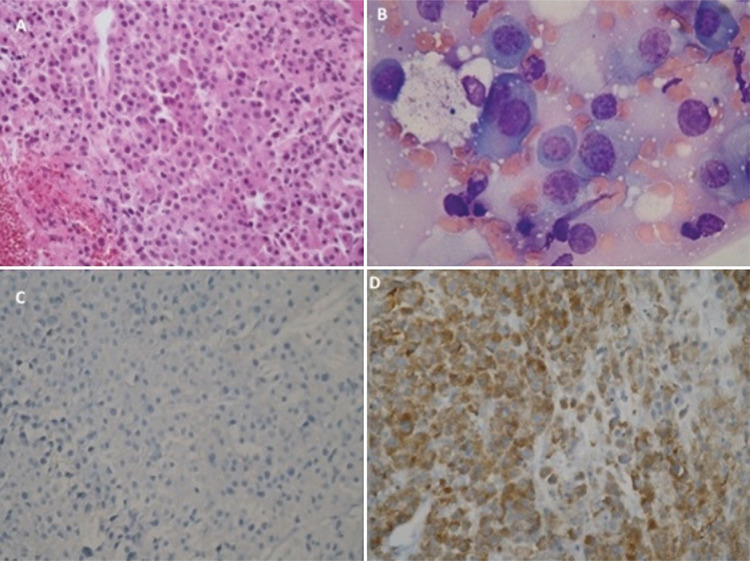
Microscopic examination revealing round monomorphic cells with vesicular and eccentric nucleus and immature plasma cells (A) (hematoxylin and eosin [H&E], 200^x^). High-power view shows plasma cells with basophilic cytoplasm, eccentric nuclei, and typical peripheral condensation of the chromatin (B) (H&E, 400^x^). Immunohistochemical staining showed that the tumor cells were negative for lambda (C) (magnification: 200^x^) and positive for kappa (D) (magnification, 400^x^).

## References

[1] Soutar R, Lucraft H, Jackson G, Reece A, Bird J, Low E, Samson D;, Guidelines Working Group of the UK Myeloma Forum; British Committee for Standards in Haematology; British Society for Haematology (2004). Guidelines on the diagnosis and management of solitary plasmacytoma of bone and solitary extramedullary plasmacytoma. Br J Haematol.

[2] Alexiou C, Kau RJ, Dietzfelbinger H, Kremer M, Spiess JC, Schratzenstaller B, Arnold W (1999). Extramedullary plasmacytoma: tumor occurrence and therapeutic. Cancer.

[3] Surov A, Holzhausen HJ, Arnold D, Schmidt J, Spielmann RP, Behrman C (2010). Intramuscular manifestation of non-Hodgkin lymphoma and myeloma: prevalence, clinical signs, and computed tomography features. Acta Radiol.

[4] Breyer RJ 3rd, Mulligan ME, Smith SE, Line BR, Badros AZ (2006). Comparison of imaging with FDG PET/CT with other imaging modalities in myeloma. Skeletal Radiol.

[5] Seval GC, Ozkan E, Beksac M (2019). PET with fluorodeoxyglucose F 18/computed tomography as a staging tool in multiple myeloma. PET Clin.

